# Comparison of a new wrist-worn accelerometer with a commonly used triaxial accelerometer under free-living conditions

**DOI:** 10.1186/s13104-018-3849-9

**Published:** 2018-10-20

**Authors:** Sachiko Sasaki, Shigekazu Ukawa, Emiko Okada, Zhao Wenjing, Tomoko Kishi, Ai Sakamoto, Akiko Tamakoshi

**Affiliations:** 1 Department of Public Health Sciences, Hokkaido University Graduate School of Medicine, Kita 15, Nishi 7, Kita-ku, Sapporo, 060-8638 Japan; 2 Department of Physical Therapy, Faculty of Human Science, Hokkaido Bunkyo University, 5-196-1 Kogane-chuo, Eniwa, 061-1449 Japan; 3 Research Unit of Advanced Interdisciplinary Care Science, Osaka City University Graduate School of Human Life Science, Osaka, Japan; 4 Department of Nutritional Epidemiology and Shokuiku, National Institutes of Biomedical Innovation, Health and Nutrition, 1-23-1 Toyama, Shinjuku-ku, Tokyo, 162-8636 Japan

**Keywords:** Accelerometer, Physical activity, Motion sensor, Assessment, Reliability, Motor activity

## Abstract

**Objective:**

The Life Microscope is a new wristband-based life recorder system that can identify various human movements. We aimed to compare physical activity data captured using the Life Microscope with data from a commonly used accelerometer.

**Results:**

Twenty-nine participants (34.6 ± 12.5 years) wore both the Life Microscope and an Active Style Pro accelerometer for 7 days. Physical activity categories were calculated by converting daily accelerometer data output into time spent at sedentary, light, moderate, and vigorous physical activity. Correlations between the physical activity category and step count data obtained from the two accelerometers were assessed using Pearson correlations, paired t-tests, intra-class coefficients, and the Bland–Altman method. Our results showed good reliability between the physical activity patterns and daily step counts obtained using both devices. Bland–Altman analysis showed good agreement between data from both accelerometers. In conclusion, both accelerometers were comparable in their measurement of step counts and time spent in different physical activity intensities under free-living conditions, and either could be used for population studies.

## Introduction

The benefits of physical activity have been well documented. Daily physical activity protects against many conditions, including cardiovascular disease [1], type 2 diabetes mellitus [2], and mortality [3, 4]. Large epidemiological studies often use self-reported assessments of daily physical activity; however, these are limited in their accuracy and may result in misclassification of data [5]. An accelerometer is an objective, small, non-invasive tool that has the potential to measure day-by-day or minute-by-minute variations in physical activity [6].

Many studies have used accelerometers attached to the hip because at this location the accelerometer can detect most major body motions, with the exception of upper limb movements [7]. However, a hip sensor must be attached to a belt or worn over clothing, which may influence wear time compliance. Recently, the National Health and Nutrition Examination Survey (NHANES) and UK Biobank studies have changed their placement of accelerometers from the traditional location on the hip to the wrist [8]. The advantages of wearing an accelerometer on the wrist include good wear time compliance. The potential of wrist accelerometers for physical activity assessment has stimulated advancements in technology so that wrist accelerometers can now detect various intensities of activity.

The Life Microscope (Hitachi Ltd., Tokyo, Japan), a new wristband-based life recorder system, utilises a tri- axial accelerometer that detects vertical, anteroposterior, and mediolateral accelerations. The Life Microscope can identify various human movements including working at a desk, eating, commuting, standing, and locomotion by detecting changes in activity; it can provide time series data in controlled environments over a 2 week period [9]. It is simple, fast, and easy to use and allows Bluetooth data transfer. Moreover, it can generate a visual representation of the wearer’s lifestyle by plotting their level of activity over the course of a day. However, no published studies have assessed whether data obtained using this new device are comparable to data obtained with conventional accelerometers in free-living conditions. The aim of this study was to compare the outputs of the Life Microscope with those of a commonly used accelerometer; the parameters assessed were daily step counts and the time spent at various activity intensities (sedentary, light, moderate, or vigorous) under free-living conditions.

## Main text

### Materials and methods

A convenience sample of 30 volunteers (15 males and 15 females) from the Hokkaido University and Hokkaido Bunkyo University student, faculty, and staff population participated in this study and 29 (15 males and 14 females) provided complete accelerometer data. Previous research comparing a new accelerometer with the Actigraph (ActiGraph Inc, USA) found a high correlation between the daily step counts obtained from both devices (r = 0.85 or greater) [10, 11]. Assuming that a high correlation could also be obtained in the current study, the required sample size when α = 0.05 and β = 0.10 is 12 people. Fifteen male and 15 female volunteers were included to take into account sex-differences in the measured values. The mean ± SD values of the study participants for age, stature, body mass, and body mass index were, respectively, 31.9 ± 10.4 years, 172.9 ± 5.7 cm, 64.4 ± 6.3 kg, and 21.6 ± 2.7 kg/m^2^ for males and 37.5 ± 14.3 years, 159.0 ± 6.1 cm, 53.2 ± 6.0 kg, and 21.1 ± 2.3 kg/m^2^ for females. Demographic and anthropometric information were recorded, and we confirmed that none of the participants had any injury or disease that would prevent them from undertaking regular physical activity. This study was conducted with the written informed consent of all participants, and was approved by the Hokkaido University Graduate School of Medicine institutional ethical board for epidemiological studies (Reference Number: 15-001).

We compared physical activity intensity categories and daily step counts obtained using the Life Microscope and the Active Style Pro HJA-750C (Omron Healthcare). The Life Microscope is an accelerometer that is worn on a wristband. It measures 21 × 39 × 15.5 mm, weighs 22 g, and records anteroposterior (x-axis), mediolateral (y-axis), and vertical (z-axis) accelerations with a resolution of 11.7 mG at 20 Hz [12]. The Life Microscope can store data for up to 14 days and data are uploaded from the device to a personal computer over a wireless network. The epoch interval for the accelerometer was set at 1 min. The Active Style Pro HJA-750C is a triaxial accelerometer that is worn on the hip. The Active Style Pro processes raw data using algorithms containing a specific equation, which have been validated with the Douglas Bag method in controlled environments [13, 14]. It measures 40 × 52 × 12 mm, weighs 23 g, and is one of the most commonly used tri-axial accelerometers for physical activity assessment [15, 16]. The Active Style Pro records anteroposterior (x-axis), mediolateral (y-axis), and vertical (z-axis) accelerations with a resolution of 3 mG at 32 Hz and has the ability to classify physical activity into locomotive and sedentary activities [13, 14]. Before assessment, each accelerometer was calibrated according to the manufacturer’s recommendations. Participants were asked to wear the Active Style Pro on the left side of their waist and the Life Microscope on their non-dominant wrist during all waking hours for 7 days, except while engaging in water activities and bathing. During the 7-day monitoring period, participants completed a daily diary to confirm their wearing time per day.

After 7 days, the data were downloaded and non-wear time was checked manually. After the initial data collection, because an error was found in the analysis software of the Active Style Pro (only for data regarding daily step counts) additional participants (11 males and 15 females) were re-recruited from Hokkaido Bunkyo University for daily step count analyses. Both accelerometers expressed the intensity of physical activity as metabolic equivalents (METs) using predictive equations [13, 17]. Physical activity patterns and the corresponding data from both accelerometers were classified into categories based on the METs for time spent at sedentary (1.1–1.5 METs), light (1.6–2.9 METs), moderate (3.0–5.9 METs), and vigorous (≥ 6.0 METs) physical activity per day [13, 18].

Statistical analyses were performed using JMP Pro version 12.2.0 for Macintosh (SAS Institute, Cary, NC). Pearson correlations and paired t-tests were used to quantify the relationship between the Life Microscope and Active Style Pro-based data. Intra-class correlation coefficients (ICC) were calculated to examine the relationship between data obtained with the Life Microscope and with the Active Style Pro. A concordance value of less than 0.60 indicates poor reliability, between 0.60 and 0.79 indicates moderate reliability, and greater than 0.80 reflects high reliability [10]. Finally, Bland–Altman plots were created to assess the level of agreement between the devices. Limits of agreement were set at ± 2 SD of the difference scores.

### Results

Data were recorded for a mean of 11.7 ± 2.6 h per day, and all participants exceeded 10 h of wear time per day. The step counts and time spent at various physical activity intensities obtained from the Life Microscope and Active Style Pro are presented in Table 1. Correlations between the data generated by the Life Microscope and Active Style Pro are presented in Table 2. There was a high correlation between the daily step counts obtained with the Life Microscope and Active Style Pro (r = 0.98, p < 0.001) and inter-monitor reliability was high (ICC = 0.98). In addition, the Life Microscope was as consistent as the Active Style Pro in its step counts (mean difference = 104.1 steps, p = 0.35) and registered 2.0% fewer steps.

**Table 1:** Daily step counts and minutes spent at various physical activity intensities as assessed by the Life Microscope and the Active Style Pro

	Life Microscope	Active Style Pro
Steps/day	5048.7 ± 2616.1	5152.8 ± 2529.7
Sedentary min/day	363.8 ± 79.4	388.5 ± 99.5
Light min/day	248.7 ± 87.3	222.1 ± 77.4
Moderate min/day	54.8 ± 34.1	86.9 ± 27.6
Vigorous min/day	9.3 ± 10.2	6.6 ± 9.6

**Table 2:** Correlations and differences between data obtained with the Life Microscope and the Active Style Pro

Life Microscope vs Active Style Pro	Pearson correlations		Paired t-tests	ICC
	r	p-value	p-value	
Steps/day	0.98	< 0.001	0.35	0.98
Sedentary min/day	0.74	< 0.001	0.06	0.72
Light activity min/day	0.92	< 0.001	< 0.01	0.92
Moderate activity min/day	0.87	< 0.001	< 0.001	0.85
Vigorous activity min/day	0.90	< 0.001	< 0.01	0.89

The correlations between the physical activity data obtained with the Life Microscope and Active Style Pro at each intensity level were moderate to high (r = 0.74, p < 0.001 when sedentary; r = 0.92, p < 0.001 for light activity; r = 0.87, p < 0.001 for moderate activity; and r = 0.90, p < 0.001 for vigorous activity). Although t-tests showed statistically significant differences between the time spent in various activity intensity categories as measured by the Life Microscope and the Active Style Pro (mean difference = 26.6 min, p < 0.01 for light activity; mean difference = 32.1 min, p < 0.001 for moderate activity; and mean difference = 2.7 min, p < 0.01 for vigorous activity), there was no difference in the time recorded as sedentary between the two monitors. Inter-rater reliability between the two monitors was also moderate to high for all physical activity categories, with ICCs of 0.72, 0.92, 0.85, and 0.89 for time spent in sedentary, light, moderate, and vigorous activity, respectively.

**Fig. 1:**
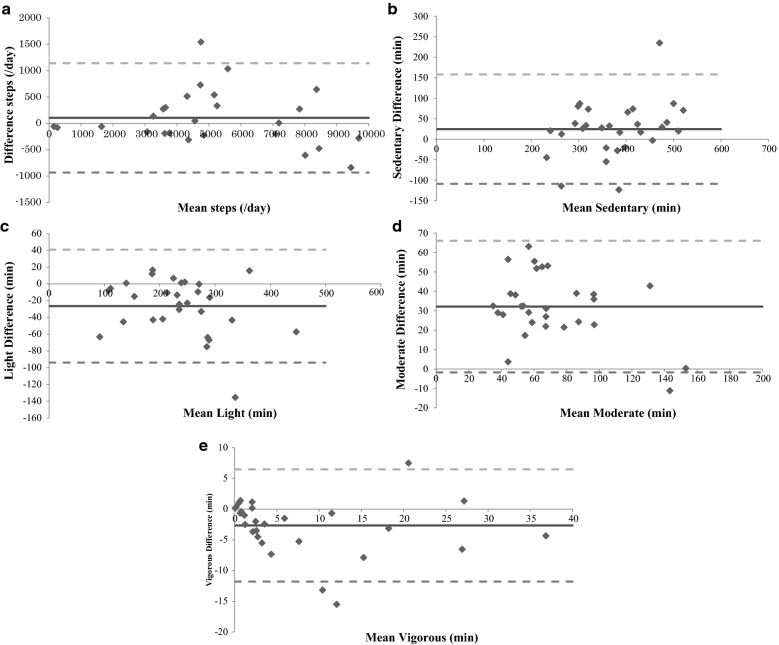
Bland-Altman plot for Life Microscope vs the Active Style Pro. Difference in step counts (**a**) and time spent at different intensities of physical activity (**b**. sedentary; **c**. light; **d**. moderate; **e**. vigorous). Solid lines: mean of difference. Dashed lines: 2 SD of difference

A Bland–Altman plot showed good agreement between the Life Microscope and Active Style Pro physical activity measurements (Fig. 1). The mean differences between the step counts and time spent in sedentary, light, moderate, and vigorous activity recorded by the two monitors were within the limits of agreement [19], and most data were within the limit of agreement bias.

### Discussion

The present study demonstrated good correlations and reliability between physical activity data obtained with a commonly used accelerometer and a new, wristband-based accelerometer in free-living conditions. Daily step counts measured by the Life Microscope were highly correlated the step counts recorded by the Active Style Pro, and the difference between the step counts from both accelerometers was small (2.0%). Such a small and consistent difference is unlikely to affect epidemiological research. Furthermore, the ICC was 98% indicating very good reliability. Therefore, we can conclude that the Life Microscope is a reliable device for measuring daily step counts. One important additional point in this study is that there was no statistically significant difference in time recorded as spent sedentary, which was the majority of the day’s activities [20], and the concordance correlation coefficient between data from the Life Microscope and the Active Style Pro was moderate. The Active Style Pro has already been confirmed to accurately separate sedentary behaviour from other activities using accelerometer-based algorithms in controlled environments and in free-living conditions [13, 14] Our results indicate that the time spent sedentary obtained by these two devices can be considered comparable.

Although there was good reliability between the data on time spent in light, moderate, and vigorous intensity physical activity obtained by the Life Microscope and the Active Style Pro, there was a significant difference between the actual times measured by the two devices. This could arise from differences in instrument sensitivity thresholds. The raw data were different depending on the accelerometer used because the magnitude of the acceleration measured depends on the electrical and mechanical properties of the measuring device [21]. Although we confirmed the separation of locomotor activities by detecting the activity change points to split out time series data in controlled environments in a previous study [9], a difference in the acceleration sensitivity thresholds may explain the discrepancies between the time spent in different activity intensities as measured by the two devices. Another reason for these differences may be the location of the accelerometer on the body. Results from previous studies suggest that the wrist may move differently than the hip during the same activity depending on what is in the hand; for example, the raw motion signature for the wrist may increase while standing holding a heavy bag or walking with a mobile phone [7, 22], which may lead to misclassification of physical activity patterns. Thus, although the results of the present study indicate that both devices are highly reliable and give well-correlated results, caution is needed when comparing outcomes and conclusions between studies that use different accelerometers.

In conclusion, our study demonstrates good correlations and reliability in the physical activity patterns and daily step counts obtained using the Life Microscope, a new wristband-based accelerometer, and the Active Style Pro in healthy adults under free-living conditions. As epidemiological surveys move toward using wristband-based accelerometers this study provides evidence that the Life Microscope is a suitable tool to use for assessment of physical activity in epidemiological research.

## Limitations

Several limitations should be considered when interpreting our results. All of the participants in our study were healthy individuals; therefore, we cannot generalize our findings to other populations. Furthermore, the number of participants was small. However, based on our sample size calculations the number of participants was sufficient to detect strong correlations between the data sets.

## Abbreviations

ICC: intra-class coefficients
METs: metabolic equivalents
